# A tailored online mindfulness intervention for junior doctors: a mixed-methods study

**DOI:** 10.1186/s12909-026-09947-5

**Published:** 2026-08-12

**Authors:** S. Moore, H. Ngo, K. Auret, A. Toffoli, L. O’Sullivan, B. Cuesta-Briand, C. Hassed, R. Chambers, N. Mavaddat

**Affiliations:** 1 Rural Clinical School of WA, University of Western Australia, Busselton, Australia; 2 Rural Clinical School of WA, University of Western Australia, Perth, Australia; 3 Rural Clinical School of WA, University of Western Australia, Albany, Australia; 4 Fiona Stanley Hospital, Perth, Australia; 5 Monash Centre for Consciousness and Contemplative Studies, Monash University, Melbourne, Australia; 6 Medical School, University of Western Australia, Perth, Australia

**Keywords:** Online mindfulness training, Junior doctors, Wellbeing

## Abstract

**Background:**

Junior doctor training is stressful and associated with significant challenges, including poor mental health and burnout. Mindfulness-based interventions (MBIs) are increasingly being offered within junior doctor education programs to support junior doctor wellbeing, with online platforms for delivery attracting interest.

**Methods:**

This mixed-method randomised waitlist control trial was conducted with junior doctors across two hospitals in Western Australia. The intervention was an 8-week online mindfulness training program, with usual training as control. Outcome measures were changes from baseline to program completion (eight weeks) and then one-month follow-up for self-reported surveys of trait mindfulness, perceived stress, self-compassion, occupational stress, and work engagement. Intervention and control participants completed surveys at all three time points. Semi-structured interviews and focus groups were conducted with participants at program completion.

**Results:**

Seventy-two (35 intervention, 37 control) junior doctors were randomised. Seventeen did not complete the baseline measures. Data for the remaining 55 participants (28 Intervention, 27 Control) were retained for analysis. Most intervention participants formally practiced mindfulness two to three times per week for five to seven minutes however quality of formal and informal practice was low. For the intervention group, perceived stress increased at program completion (-1.4, 95% CI − 2.6, -0.3, *p* = 0.0151) while work engagement was decreased (-2.9, CI − 5.6, -0.2, *p* = 0.0355) at final follow-up. There were no significant changes in any of the outcomes for the control group at any follow-up point. Thirteen intervention participants undertook focus groups or individual interviews about their experience. Despite lack of positive quantitative findings, benefits reported regarding the program during interview included: improved self-awareness, emotion regulation, self-compassion and work productivity, as well as professional and personal relationships. Barriers to participation in the program included limited time, work stress, and challenges with elements of the intervention.

**Conclusion:**

This novel online mindfulness training program was not associated with improvements in measured outcomes including trait-mindfulness, perceived stress, self-compassion, occupational stress or work engagement for participating junior doctors. This contrasts with our qualitative findings suggesting that junior doctors experience some wellbeing benefits from participating, and with the results of our similar trial in medical students. While the dose of mindfulness practice was the same for both trials, junior doctors’ adherence to practice was lower than medical students’ adherence. Organisational factors such as stressful workplaces and time limitations likely minimised the impact of the mindfulness intervention in this group.

**Open Science:**

Trial Registry: Australian New Zealand Clinical Trials Registry (ANZCTR).

Trial Registration ID: ACTRN12622001248752.

Protocol and statistical analysis plan: https://anzctr.org.au/.

Request ID: 384649.

Date of Registration: 16th September 2022.

Data sharing: De-identified participant data, data dictionary, analytical code used to process data and materials associated with the intervention may be shared with researchers providing a methodologically sound proposal on a case-by-case basis at the discretion of the Primary Sponsor.

Funding and conflicts of interest: Funding was provided by University of Western Australia and Fiona Stanley Hospital. There are no conflicts of interest to declare.

## Background

The first few years of junior doctor training have been recognised as a particularly stressful period in a doctor’s career [1, 2] with significant impact on both the mental health [3–5] and work efficiency of junior doctors [6]. Burnout during early stages of junior doctor training has negative effects on trainees’ professional behaviour and impacts patient safety through poor communication and high medical error rates [7–9]. Further, personal impacts of burnout such as depression [10, 11] and suicidal ideation [12] are worrisome, as many Australian doctors, particularly junior doctors, do not seek help due to concerns regarding privacy, confidentiality, feelings of humiliation, and fear of being reported to medical registration bodies [13]. Underlying reasons for junior doctor stress and burnout have been well documented in the literature and include poor work-life balance, anxieties about suboptimal practices and poor patient outcomes, high workloads and on-call commitments, low job satisfaction, poor career development and poor work environment [14].

In response to these significant problems for junior doctors worldwide, statements have been issued by national medical associations recommending evidence-based change at national, hospital, program and non-work levels to optimise junior doctor wellbeing. These organisations include the Collaborative for Healing and Renewal in Medicine (CHARM) - a group of experts in medical education and burnout interventions based in the United States [15], the British Medical Association in the United Kingdom [16] and the Australian Medical Association in Australia [17]. The list of wellbeing interventions recommended by most of these bodies, including CHARM, incorporate system-level changes as well as programs aimed at the individual, including the development and research of evidence-based mindfulness and resilience training for medical students and trainee doctors for best practice [15].

Meta-analyses of studies investigating the effectiveness of mindfulness-based interventions (MBIs) for reducing the symptoms of depression, anxiety and stress in patient populations with psychological problems report positive results [18, 19]. MBIs have also been found to increase the trait of self-compassion amongst non-clinical populations [20, 21]. Higher levels of self-compassion are associated with lower levels of stress and more effective coping strategies [22], better emotion regulation in times of adversity [23] and protection from burnout [7]. Additionally, studies have demonstrated that mindfulness practice may improve engagement with work [24, 25], and higher levels of work engagement are generally regarded as an antidote to burnout [26].

In line with the CHARM and other recommendations, MBIs have been introduced to medical school curricula around the world and their effectiveness for reducing psychological distress and promoting wellbeing in medical students has been documented [27, 28]. MBIs have also been taken up by hospital medical education units to provide junior doctors with tools to manage their stress, optimise their wellbeing and reduce the risk of burnout. Quantitative studies evaluating these interventions have demonstrated improvements in wellbeing outcomes for junior doctors, including in objective measures such as trait-mindfulness, perceived stress, mood, self-compassion and burnout [29–34]. Qualitative and mixed-methods studies have also assessed MBIs delivered to junior doctors, reporting their utility for stress-management at work, improved self-care and enhanced relationships [30, 35–38].

Nevertheless, systematic reviews of MBI studies have not been entirely consistent, with objective effects of these interventions on junior doctor wellbeing and burnout not always observed. It has been suggested that this is mainly due to the methodological limitations of the primary studies (such as study design and sample size) but may also represent limited impacts of the interventions themselves in the junior doctor cohort [39–41].

Possible reasons for the limited impact of such programs may relate to the mode of delivery of the interventions. Traditional Mindfulness-Based Stress Reduction programs make up the majority of MBIs introduced to junior doctors at hospitals in studies. These usually involve between one to 2.5 h per week of face-to-face training over approximately eight weeks [39–41]. Junior doctor participants in studies of such face-to-face MBIs however report challenges with attendance due to conflicting clinical commitments [33] and inadequate support for ‘protected time’ to attend sessions [42, 43]. They may also not feel comfortable openly discussing challenges and mental health topics in a group of their peers. Despite reports that junior doctor working hours have improved over the past few decades, this may paradoxically increase pressure on junior doctors to complete their work in a shorter period [3] making it even more challenging to participate in intensive mindfulness training programs.

Delivering mental health support via online platforms, including mindfulness training, has therefore become increasingly popular for health care professionals, including junior doctors [44, 45]. Online wellbeing programs are accessible, offer flexibility, and teach health practitioners practical skills that can be implemented immediately into practice [44, 45]. Less intensive programs offering short, face-to-face workshops [46, 47], very brief 10-day interventions [48] and four-to-six-week programs using mobile mindfulness apps with no facilitator contact [49–52] as well as facilitated teaching sessions via Zoom [53–55], have been appraised with junior doctors. However, most were single arm studies, with only one bespoke online MBI for health practitioners evaluated in a randomised control trial [50]. Study measures have included various outcomes such as burnout, trait mindfulness, self-compassion, mood and stress [46–48, 50–55] as well as qualitative evaluation [49, 53–55]. Although these studies suggest that both brief workshops and online programs that teach mindfulness skills to junior doctors are associated with positive trends in wellbeing, several limitations have been identified, including small sample sizes, lack of control groups, high dropout rates and selection bias. The RCT evaluating an online MBI of 3-months duration reported a significant increase in self-compassion and mindfulness from baseline to post-intervention within the intervention group, with improvements in self-compassion maintained at 6-month follow-up, with no changes reported in the control group at either follow-up time point [50]. This finding warrants further high-quality research to strengthen the evidence for the efficacy of online MBIs at improving wellbeing in junior doctors [39–41]. As tailored online mindfulness training programs for health care professionals have been associated with lower dropout rates from trials and may have greater potential for effectiveness amongst junior doctors, priority should be given to interventions specifically designed for this group [44]. Of note, there are currently no studies conducted with junior doctors that directly compare the efficacy of in-person and online MBIs.

We have previously reported on the positive findings of a feasibility pilot study and randomised control trial evaluating the effects of a tailored 8-week online mindfulness training program on wellbeing outcomes in medical students [56, 57]. The aim of this study was to assess the effectiveness of the same tailored 8-week online mindfulness training program in reducing junior doctors’ perceived and occupational stress levels, and improving their trait-mindfulness, self-compassion, and work engagement, in comparison to a control group, who only undertook the normal junior doctor training curriculum. The focus groups and individual interviews aimed to understand the experiences of junior doctors during the online MBI, their perceptions of the benefits of the intervention with regards to their wellbeing and work engagement, and the difficulties they encountered during the 8-week intervention. We anticipated that online mindfulness training would be a valuable tool for stress management, wellbeing, and professional development for junior doctors at the commencement of their hospital training.

## Methods

### Study design

**Fig. 1:**
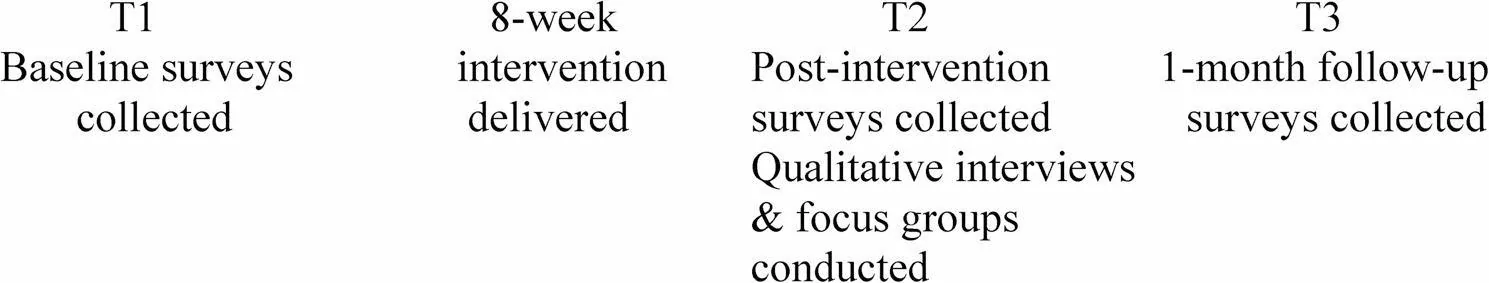
Study Timeline

This was a mixed-methods evaluation of a randomised waitlist control trial of individual participants with a 1:1 allocation ratio based on a superiority framework. There were no changes to the original protocol after the trial commenced. See the CONSORT Flow Diagram (Fig. 1) for an overview of the study design. The qualitative part of the study involved conducting focus groups or individual interviews with a subset of trial participants, who were sampled using a convenience method via an email invitation and follow-up telephone call.

### Setting

The trial was conducted within the South Metropolitan Health Service (SMHS), including Fiona Stanley Hospital (FSH), a large tertiary hospital, and Fremantle Hospital (FH), a secondary hospital, both located in Perth, Western Australia.

### Participants

Inclusion criteria: all interns (first year post-graduation) and resident medical officers (post-graduate doctors, predominantly not yet on a specialty training program) employed by SMHS during the years of 2022 and 2023, aged 18–60 years. Exclusion criteria: medical students, registrars (specialty-trainees) and consultants.

### Ethics approval and consent to participate

This study was approved by SMHS Human Research Ethics Committee (Ref RGS0000005076). All participants provided informed, written consent to participate prior to commencement of the study.

### Intervention & comparator

The intervention was a tailored, 8-week online Mindfulness Training Program (referred to as the MTP) in addition to the usual junior doctor curriculum. The comparator was the usual junior doctor curriculum alone. This ‘waitlist’ control group were guaranteed access to the MTP on completion of the study. The comparator condition was chosen for pragmatic reasons, as this was a low-budget trial with limited resourcing. The MTP was originally developed for medical students by the coordinating principal investigator, SM, an experienced mindfulness practitioner and medical educator, with input from two mindfulness experts, CH and RC, from Monash University (Moore et al., 2020; Moore et al., 2024). The program was adapted for use by junior doctors by SM and a medical education registrar at FSH, LO, and involved making small modifications to the original MTP to ensure that the lectures were relevant to the junior doctor context with provision of information describing the applications of mindfulness relevant to work rather than its study-related applications.

The MTP provides junior doctors with short, regular mindfulness teaching videos and meditation practices in order to minimise time spent engaging with the program content within their busy schedules, while encouraging them to complete the mindfulness training daily. The coordinating principal investigator SM is also the program facilitator (PF).

The MTP in the study was administered electronically by the FSH Medical Education Unit (MEU) via weekly emails and daily messages using the WhatsApp mobile application over an 8-week period from March to May to two cohorts of junior doctors in 2022 and 2023. It includes eight mini-lectures (10-minutes duration each) focusing on various applications of mindfulness relevant to junior doctors and eight guided mindfulness meditation practices (five-minutes duration each) related to the mini-lecture content. Specific content of the mini-lectures and mindfulness practices is shown in Table 1.

The mini-lecture topics and mindfulness meditation practices were chosen to train specific mindfulness skills. The first four weeks of the program focused on the development of attention regulation, such as observing and acting with awareness, while the latter four weeks focused on attitudinal and affective skills, such as non-judging, non-reactivity and acceptance.

**Table 1:** Content of the MTP

Domains	Mini-lectures	Mindfulness meditation practices
Attention Regulation	Week 1. Multi-tasking versus effective task switching	Week 1. Body scan
	Week 2. Stress reduction and performance	Week 2. Mindful breathing
	Week 3. Reducing distraction and procrastination	Week 3. Thought-labelling
	Week 4. Mindful communication	Week 4. Mindful listening
Attitudinal & Affective Skills	Week 5. Regulating emotions	Week 5. Working mindfully with emotions
	Week 6. Compassion	Week 6. Loving kindness
	Week 7. Self-compassion	Week 7. Self-compassion
	Week 8. Mindful use of technology	Week 8. Mountain meditation

One mini-lecture was emailed as an audiovisual recording to participants at the beginning of each week and one mindfulness meditation was sent via a WhatsApp group as an audio recording daily from Monday to Friday over the 8-week program. The main behaviour change techniques, according to the Behaviour Change Technique Taxonomy v1, utilised in the online MTP include (i) repetition to assist with habit formation, with daily reminders to practice formal mindfulness; (ii) feedback and monitoring via self-monitoring of behaviour using the MAQ delivered weekly to participants via email and monitoring of outcome measures collected via email at program completion and one-month follow-up; and (iii) shaping knowledge via the delivery of the weekly mini-lectures, which provided context for the application of mindfulness in the participants’ daily lives [58].

### Data collection

#### Outcome measures & instruments

The primary outcome measures were changes in trait-mindfulness (Frieberg Mindfulness Inventory, FMI) [59], perceived stress (Perceived Stress Survey, PSS) [60], self-compassion (Self-Compassion Scale, SCS) [61], occupational stress (Senior Doctors Stress Inventory, SDSI) [62] and work engagement (Utrecht Work Engagement Scale, UWES) [63] from baseline (T1) to program completion 8-weeks later (T2).

The secondary outcome measures were changes in the same five measures from baseline (T1) to the final follow-up one-month after program completion, which was approximately three months after baseline (T3).

These outcome measures were chosen to assess the impact of the intervention as they have been frequently reported in other studies of MBIs delivered to medical students and junior doctors, allowing for comparisons to be made. Multiple measures were required as there is no consistent evidence that one measure is more important than another in evaluating outcomes in mindfulness interventions [40, 64–66]. The FMI was chosen to measure attention regulation and meta-awareness, the PSS and SDSI were selected to measure emotion regulation and reactivity, the SCS was chosen to measure self-compassion, and the UWES was selected to measure focus, emotion regulation and task engagement.

Other data collected at T1 via email survey included demographics (age, gender, ethnicity, postgraduate year), details of any prior training in mindfulness, whether participants already had a regular mindfulness practice and a measure of their current psychological distress via the K-10 survey [67]. Adherence to the MTP was measured using the Mindfulness Adherence Questionnaire (MAQ) [68], which records the frequency, duration, and quality of formal and informal mindfulness practice during the previous week. This was administered weekly to intervention participants via email between T1 and T2. At T2, control participants were asked to report the frequency and duration of any self-directed formal mindfulness practice they had undertaken during the previous eight weeks. At T3, all participants were asked to report on the frequency and duration of any mindfulness practice they had undertaken between T2 and T3. Figure 1 illustrates the timeline for the study.

The qualitative component of the study involved individual interviews and focus groups conducted with intervention group participants. Participants were originally invited to join a focus group. For those who could not attend the focus group sessions, individual interviews were then offered. The collection of qualitative data both via individual interviews and focus groups has been used in other studies of MBIs conducted with junior doctors [35, 54]. The interview questions were designed to explore junior doctors’ experience of participating in an online MBI and to gain a deeper understanding of the quantitative findings including individual and systemic issues impacting on their engagement with the MTP.

Key features of the instruments used are summarized in Table 2.

**Table 2:** Study instruments

Instrument	Description
*The Kessler Psychological Distress Scale* (K-10) [67]	The K-10 was used to measure psychological distress at baseline. This scale was administered as part of the intake survey to screen for participants at high risk of psychological distress. The K-10 is a widely used, validated and reliable tool used for measuring the severity of symptoms of anxiety and depression an individual has been experiencing in the previous four weeks. Participants respond to each item using a 5-point scale ranging from 1 (none of the time) to 5 (all the time). Scores range from 10 (minimum) to 50 (maximum). Higher scores indicate a higher likelihood of anxiety or depression [67].
*Freiburg Mindfulness Inventory* (FMI) [59]	The FMI is a validated and reliable 14-item instrument to measure trait mindfulness. Responses are given on a scale ranging from 1 (rarely) to 4 (almost always). Total possible scores range from 14 to 56; higher scores indicate higher levels of mindfulness [59].
*The Perceived Stress Scale* (PSS) [60]	The PSS was used to measure perceived stress. The PSS is a widely used, validated and reliable 10-item instrument, which measures the degree to which situations in an individual’s life are perceived as stressful. Participants respond to each item using a scale ranging from 0 (never) to 4 (very often). Total possible scores range from 0 (minimum) to 40 (maximum); higher scores indicate higher levels of perceived stress [60].
*The Self-Compassion Scale* (SCS) [61]	The SCS was used to measure self-compassion. The SCS is a validated and reliable 26-item instrument that assesses components of self-compassion. Responses are given on a scale ranging from 1 (almost never) to 5 (almost always). Total possible scores range from 26 to 130; higher scores indicate higher levels of self-compassion [61].
*The Utrecht Work Engagement Scale* (UWES) [63]	The UWES was used to measure work engagement. The UWES is a validated and reliable 17-item instrument to assess engagement with their work. Responses are given on a scale ranging from 0 (never) to 6 (always/every day). Total possible scores range from 0 to 102; higher scores indicate higher levels of work engagement [63].
*Specialist Doctors Stress Inventory* (SDSI) [62],	The SDSI was used to measure occupational stress. The SDSI is a validated and reliable 25-item self-report instrument measuring four types of occupational stressors: clinical responsibility, demands on time, organizational constraints and a lack of personal confidence. Each item is rated on a 5-point Likert-type scale ranging from 0 (never) to 4 (very often). Scores range from 0 (minimum) to 100 (maximum). A higher total score indicates a higher level of perceived occupational stressors [62]. This is the only validated scale available for measuring occupational stress in doctors.
*The Mindfulness Adherence Questionnaire* (MAQ) [68]	The MAQ is a newly developed, validated and reliable 12-item self- report of adherence to mindfulness-based practice within the past week. The first two items measure quantity of formal practice in terms of frequency and average duration (in minutes). The final ten items measure the quality of mindfulness practice, both formal and informal. Practice quality is the level to which a person is practising mindful attention and attitude during meditation and informal practice. Items are scored on a 7-point Likert- scale ranging from 0 (never) to 6 (always). Total possible scores for the quality of formal and informal practice range from 0 to 24 and 0 to 36 respectively. Higher scores reflect higher practice quality [68].

### Participant safety

Participants were asked to complete the Kessler Psychological Distress Scale (K-10) [67] at baseline as part of the intake survey to screen for participants at high risk of psychological distress. Participants (*n* = 8) who recorded a score of 30 or higher (highest score 39 in this sample) were advised that their score was in the very high range and were provided with contact details for local mental health support hotlines and employer counselling support services as per the research team’s duty of care. These participants were encouraged to seek medical care if required. No specific harms monitoring was conducted during the course of the intervention. However, there were no adverse effects reported to the study team, nor emerged in the qualitative data, and no participants withdrew from the study.

### Qualitative data collection

On completion of the 8-week intervention, intervention group participants were invited to participate in focus groups or individual interviews, depending on their availability. Focus groups consisted of two to four participants. The interviews were semi-structured, with open-ended questions. Questions related to participants’ experiences during the 8-week intervention, including perceived benefits from participating in the MTP and any difficulties they encountered that impacted on their participation. The interview guide is presented in Appendix 1.

Interviews were conducted by SM in June 2022 and 2023 using Microsoft Teams video call facility. Research assistant RB was present for most of the individual interviews and focus groups to provide research support. Following each focus group and individual interview, SM made notes of her reflections to assist with navigating successive qualitative data collection. There was no need for any repeat interviews with any of the participants. Audio-visual recordings were utilised to collect data and transcribed verbatim by SM using Microsoft Teams software. Transcripts were not returned to participants for comment or correction.

### Recruitment

Interns and resident medical officers employed by the SMHS were invited to participate in the study during a two-month period from January to February in two successive years; Cohort One in 2022 and Cohort Two in 2023. The FSH MEU notified all interns and resident medical officers about the study during Orientation Week presentations in January of each intake year then emailed a Participant Information and Consent Form (PICF), inviting them to enroll in the study. In addition, promotional flyers were placed on hospital grounds, encouraging junior doctors to enroll in the study. Junior doctors were informed that participation was voluntary, would have no impact on their employment status or performance reviews, and that they could withdraw at any time. For the interviews and focus groups, consenting participants were contacted via email and phone to confirm their availability and to arrange a time to participate at the completion of the 8-week intervention. All participating junior doctors within the trial were required to sign informed consent.

### Sample size

A planned sample size of a minimum of *n* = 30 in each group was expected to be adequate to afford a high level of statistical power (around 0.9) to detect a difference in levels of improvement, for intervention participants versus control participants, on the outcome measures (PSS, SCS, FMI, SDSI and UWES) at a statistical significance level of α = 0.01. The hypothesized improvement was estimated to be modest for intervention participants (i.e., of a magnitude around 9%-15% of the participants’ baseline measure), compared to negligible change (around 1%-5%) for control participants.

### Randomisation

Randomisation codes were generated using the PROC PLAN procedure in SAS after each yearly (2022 and 2023) recruitment was completed. Randomisation was run separately for male and female participants, as a ‘surrogate’ for stratification, due to (a) previous evidence for differential effects of mindfulness training in each gender [69] and (b) disproportionately more female participants in our sample. Blinding of participants to the investigator (SM) and the statistician (HN) was not feasible in this RCT due to the weekly nature of the intervention provision and weekly follow-up via the MAQ for the Intervention group over the course of eight-weeks, versus follow-up at only T2 and T3 for the Control group.

### Data analysis

#### Quantitative data analysis & statistical methods

Study data were collected and managed using the REDCap electronic data capture application hosted at the University of Western Australia [70, 71]. Data were then exported to Excel for cleaning and then to SAS (version 9.4) software for statistical analyses. Consistent with the randomised controlled design, an intention-to-treat (ITT) analysis approach was followed. To gain a deeper insight into the potential impact of missing data due to loss-to-follow up, a sensitivity analysis was also conducted for the four primary outcome measures, comparing results from the ITT analytical approach to the per-protocol approach. Statistical significance was set at α = 0.05.

Data on outcome measures were first summarised descriptively (i.e., frequency count and percentage for categorical variables, and mean and standard deviation for continuous variables). The statistical significances of before-after within-group score changes on trait- mindfulness, perceived stress, self-compassion, occupational stress and work engagement were assessed using paired t-test. Measures of mindfulness practice for intervention participants were depicted descriptively via boxplots. Statistical significance of between-group difference in score changes from T1 to T2 was assessed using generalized linear modelling (GLM).

### Qualitative analysis

Qualitative data were subjected to thematic analysis [72]. To increase familiarisation with the data, SM transcribed all interviews verbatim, re-read the transcripts, and maintained reflection notes to record personal interpretations of the data. SM identified codes using an inductive process aided by the NVivo 2022 software, which were organized into categories using an iterative process.

NM and HN also independently coded a subset of the interviews and focus groups. SM, NM and HN then reviewed the codebook and together developed a thematic framework. Themes most relevant to the research questions of participant experience, particularly the barriers and benefits of the intervention, were then discussed with another member of the research team (KA) before coming to a final agreement. Participants were not asked to provide feedback on the findings.

### Research team positionality

SM identifies as a white, heterosexual, cisgender woman who has lived experience as a medical student, junior doctor and mindfulness practitioner. She has worked as a senior lecturer and researcher at a Western Australian university in a rural town for twelve years. She undertook her medical school training at a Western Australian university, including an extended rural placement, and completed her early junior doctor training at a tertiary hospital in Western Australia. She is also a long-term mindfulness practitioner and is currently completing her PhD evaluating the impact of online mindfulness training on medical student and junior doctor wellbeing. SM had no prior relationship with any of the junior doctor participants prior to commencing the study. The participants were informed that SM was a clinician and medical educator conducting the study as part of her PhD research. NM is a female medical practitioner, senior medical educator and researcher with an interest in wellbeing and neuroscience at the University of Western Australia (UWA) and SM’s primary coordinating PhD supervisor. HN is a female research fellow and biostatistician at UWA and one of SM’s coordinating PhD supervisors. KA is a female medical practitioner, senior medical educator and researcher with an interest in palliative care and wellbeing at UWA and one of SM’s coordinating PhD supervisors. As described below, codes, categories and themes were discussed with NM, HN and KA, at each stage of the iterative process of analysis. SM prepared the manuscript, with feedback and input provided initially by NM, HN and KA, and later by the wider research team. CH is a general practitioner, medical educator and researcher with extensive experience in delivering mindfulness training within medical curricula who provided advice and mentorship. RH is a clinical psychologist and expert in mindfulness, particularly in tertiary settings and via online delivery. AT is an emergency physician and director of clinical training at FSH. LO is a physician trainee and medical education registrar at FSH. BCB is a Research Fellow with the RCSWA with expertise in qualitative methods.

### Reflexivity

As a clinician researcher, medical educator and mindfulness practitioner who has worked as a junior doctor earlier in her training, SM has a deep understanding of, and personal interest in, the participants’ issues and experiences. SM was an insider to the experience of working as a junior doctor in a busy tertiary hospital, and training in mindfulness as a general practitioner. NM and KA are also clinicians with a previous lived experience of working as junior doctors in the Western Australian hospital setting. HN is more of an outsider to the experiences of junior doctors working in the tertiary hospital environment. Throughout the study, collectively we worked as both insiders and outsiders with an awareness of the potential for our own lived experiences and cultural biases to influence our interpretations of the junior doctors’ experiences to ensure our analysis was a true reflection of their experiences.

## Results

### Participant flow

**Fig. 2:**
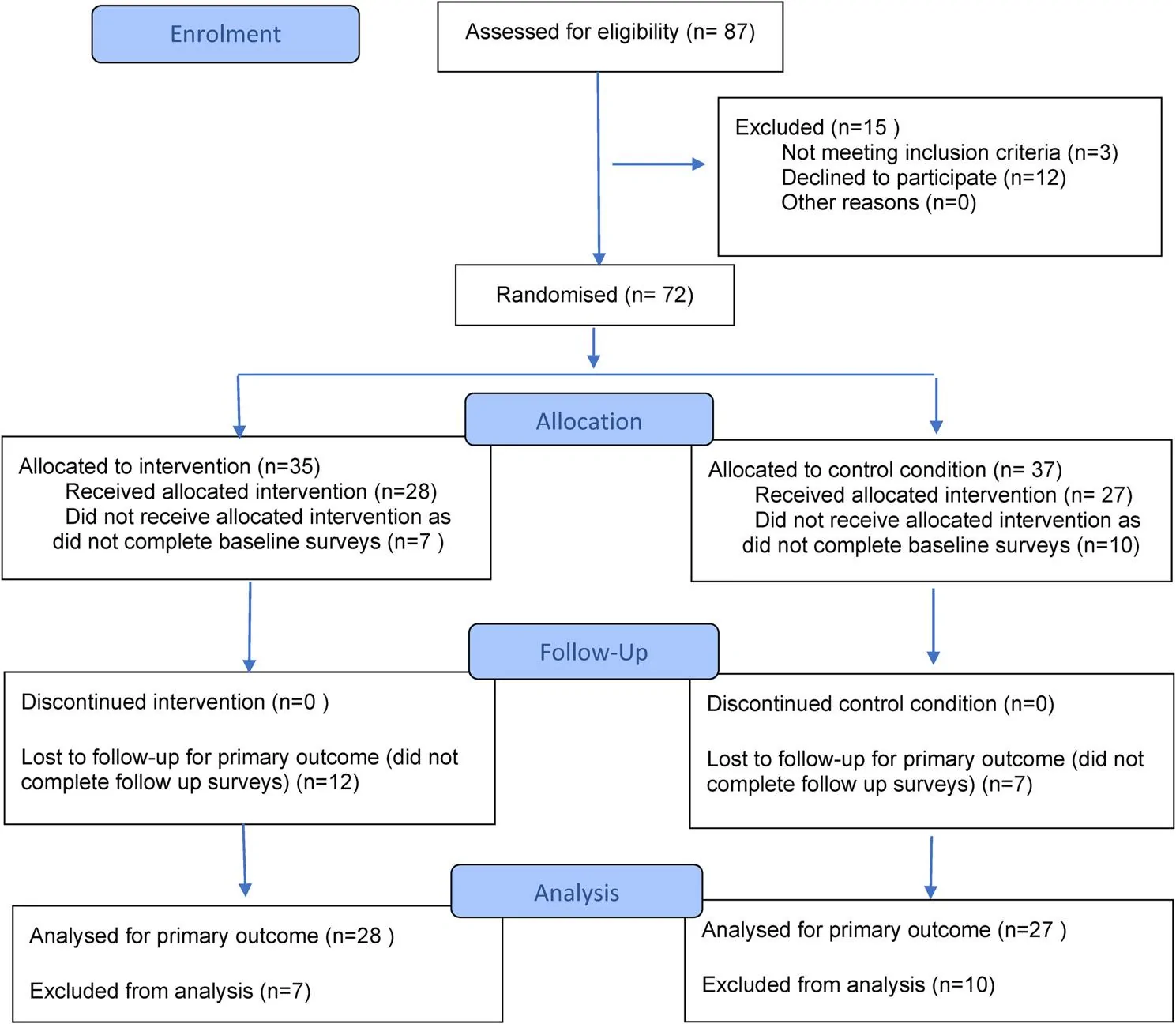
CONSORT 2025 Flow Diagram

An overview of participant flow through the study is presented in Fig. 2A total of 72 junior doctors were assessed as eligible to participate in the RCT and were randomised, 35 to the intervention and 37 to the control arm. Seven intervention arm participants and ten control arm participants did not complete baseline measures, so were excluded from all analyses. Twenty-eight intervention arm participants and 27 control arm participants completed baseline measures and were included in the analysis. Participants who did not return follow-up data at program completion (T2) and/or final follow up (T3) were considered lost to follow-up, although their data were still included in analyses, as per the ITT approach, with missing data at follow-up imputed to denote “zero” change compared to baseline.

Thirteen intervention participants consented to take part in semi-structured interviews.

### Baseline data

The statistics in Table 3 show that the two groups were relatively comparable across most key characteristics at baseline. Female participants made up 62% of the study population. Junior doctors in their first postgraduate year (PGY1) made up 82% of the cohort, with more PGY1 participants in the intervention group than the control group (*p* = 0.0446). 60% of participants were Caucasian. One-fifth of the cohort reported prior exposure to mindfulness practice before commencing the program. At baseline, most participants across the two groups reported a moderate level of psychological distress according to their mean K10 scores (mean = 20.1 points) [67]. Table 3 also shows that the intervention and control groups returned comparable scores for the perceived stress score, self-compassion score, trait-mindfulness score, work engagement score and occupational stress score. The internal consistency reliability (Cronbach alpha) was high for each of these scales: 0.908 for K10, 0.882 for PSS, 0.959 for SCS, 0.909 for FMI, 0.915 for UWES, and 0.928 for SDSI.

**Table 3:** Description of the two randomised groups at baseline

Characteristics		Intervention (*n* = 28)		Control (*n* = 27)		
		Mean	SD	Mean	SD	*p*-value
*Age (in years) †*		*25.7*	*3.3*	*27.2*	*4.0*	*0.1573*
*K10 score †*		*19.1*	*5.8*	*21.0*	*7.9*	*0.3056*
*PSS score †*		*15.1*	*6.3*	*17.4*	*6.3*	*0.1831*
*SCS score †*		*84.6*	*20.7*	*77.1*	*18.2*	*0.1628*
*FMI score †*		*33.4*	*8.9*	*31.5*	*8.1*	*0.4091*
*UWES score †*		*65.0*	*12.3*	*64.1*	*14.4*	*0.8058*
*SDSI score †*		*51.7*	*16.8*	*52.7*	*21.3*	*0.8380*
		Frequency	%	Frequency	%	p-value
Gender	Male	11	39%	10	37%	0.8638
	Female	17	61%	17	63%	
Ethnicity	Caucasian	15	54%	19	70%	0.2028
	Non-Caucasian	13	46%	8	30%	
PGY	1	26	93%	19	70%	**0.0446**
	2 to 6	Suppressed	Suppressed	8	30%	
Prior mindfulness	Yes	7	25%	Suppressed	15%	0.3497
	No	21	75%	23	85%	
Year of Recruitment	2022	19	68%	18	67%	0.9251
	2023	9	32%	9	33%	

Other (categorical) variables are presented in frequency and percentage, with between-group comparisons conducted using logistic regression. Small counts (< 5) are suppressed

*PGY* Postgraduate year,* K-10* Kessler Psychological Distress Scale (10-item version), *PSS* Perceived Stress Scale, *SCS* Self Compassion Scale, *FMI* Freiburg Mindfulness Inventory, *UWES* Utrecht Work Engagement Scale, *SDIS* Senior Doctors Stress Inventory

† Continuous variables, in italics, are presented in mean & standard deviation (SD), with between-group comparisons conducted using independent two sample t test

### Primary outcomes

Table 4 shows the intervention participants’ mean PSS scores were significantly worse at T2 compared to T1 (estimate − 1.4, 95% CI -2.6, -0.3; *p* = 0.0151). Intervention group participants’ SCS, FMI, UWES and SDSI scores did not change significantly at T2 compared to T1. There were no significant changes within control group participants in any of the outcome scores at T2 compared to T1. Additionally, there were no between-group differences in score changes from T2 compared to T1 for any of the outcomes (Table 5).

Results from the sensitivity analysis, comparing the intention to treat versus per protocol analytical approaches, are shown in Appendix 2. The two analytical approaches converged.

### Secondary outcomes

Table 4 also shows that intervention group participants’ UWES scores decreased at T3 compared to T1 (estimate − 2.9, CI -5.6, -0.2; *p* = 0.0355). Intervention group participants’ PSS, SCS, FMI and SDSI scores did not change significantly at T3 compared to T1. There were no significant changes within control group participants in any of the outcome scores at T3 compared to T1. Additionally, there were no between-group differences in score changes from T3 compared to T1 for any of the outcomes (Table 5).

**Table 4:** Scores on Perceived Stress Scale (PSS), Self-Compassion Scale (SCS), Freiburg Mindfulness Inventory (FMI), Utrecht Work Engagement Scale (UWES), and Senior Doctors Stress Inventory (SDSI) at T1, T2 and T3

Group	Scale	T1		T2		T3		Difference: T2-T1 ‡		Difference: T3-T1 ‡	
		mean	SD	mean	SD	mean	SD	estimate	*p*-value	estimate	*p*-value
Intervention	PSS	15.1	6.3	16.5	6.0	16.4	6.2	-1.4	0.0151 *	-1.3	0.1240
(n_I_=28)	SCS	84.6	20.7	82.0	17.8	85.1	17.9	-2.6	0.0826	0.5	0.7828
	FMI	33.4	8.9	34.1	8.5	34.0	8.5	0.7	0.1954	0.6	0.3951
	UWES	65.0	12.3	63.7	14.4	62.1	12.8	-1.3	0.4432	-2.9	0.0355 *
	SDIS	51.7	16.8	51.7	17.5	50.4	15.9	0.0	1.0000	1.3	0.4873
Control	PSS	17.4	6.3	17.7	6.0	18.7	6.4	-0.3	0.6943	-1.3	0.1739
(n_C_=27)	SCS	77.1	18.2	74.9	17.7	76.3	18.4	-2.2	0.0792	-0.8	0.6250
	FMI	31.5	8.1	32.5	8.5	30.8	8.0	1.0	0.2431	-0.7	0.3235
	UWES	64.1	14.4	62.9	14.6	62.8	16.6	-1.2	0.4764	-1.3	0.5124
	SDIS	52.7	21.3	52.0	16.2	52.7	20.0	0.7	0.6115	0.0	0.9748

‡ The three scales SCS, FMI, and UWES measure ‘positive’ constructs; therefore a ‘desired’ change would be an increase in score from T1 to T2 or to T3

By contrast, the PSS and SDIS scales measures a ‘negative’ construct; therefore a ‘desired’ change would be a decrease in score from T1 to T2 or to T3

Within-group score differences, or changes, for all the five outcome measures have been calculated such that numerically positive differences reflect ‘desired’ changes

All statistics are calculated using the intention-to-treat approach

* Indicates effect significant with *p* < 0.05

**Table 5:** Between-group differences in score changes, for Intervention (I) vs. Control (C) groups, from T1 to T2 and T3 respectively, on Perceived Stress Scale (PSS), Self Compassion Scale (SCS), Freiburg Mindfulness Inventory (FMI), Utrecht Work Engagement Scale (UWES), and Senior Doctors Stress Inventory (SDSI)

	Difference: I v C (T2-T1)				Difference: I v C (T3-T1)			
	estimate	LL	UL	*p*-value	estimate	LL	UL	*p*-value
PSS ‡	-1.1	-3.1	0.9	0.2765	0.1	-2.5	2.6	0.9470
SCS	-0.4	-4.2	3.5	0.8561	1.4	-3.6	6.3	0.5895
FMI	-0.3	-2.3	1.8	0.8042	1.3	-0.7	3.3	0.1954
UWES	-0.1	-4.8	4.7	0.9787	-1.6	-6.2	3.0	0.4784
SDSI ‡	-0.7	-5.3	3.8	0.7331	1.3	-3.2	5.7	0.5663

‡ The three scales SCS, FMI, and UWES measure ‘positive’ constructs; therefore a ‘desired’ change would be an increase in score from T1 to T2 or to T3

By contrast, the PSS and SDIS scales measures a ‘negative’ construct; therefore a ‘desired’ change would be a decrease in score from T1 to T2 or to T3

Following on from Table 4 above, where numerically positive within-group score changes indicate ‘desired’ effects, negative between-group differences here in Table 5 would denote inferiority for the intervention group, compared to the Control group. Note, however, none of the comparisons in Table 5 is statistically significant

*All statistics are calculated using the intention-to-treat approach*

*LL* Lower Limit & *UL* Upper Limit, for 95% Confidence Interval

### Intervention adherence

**Fig. 3:**
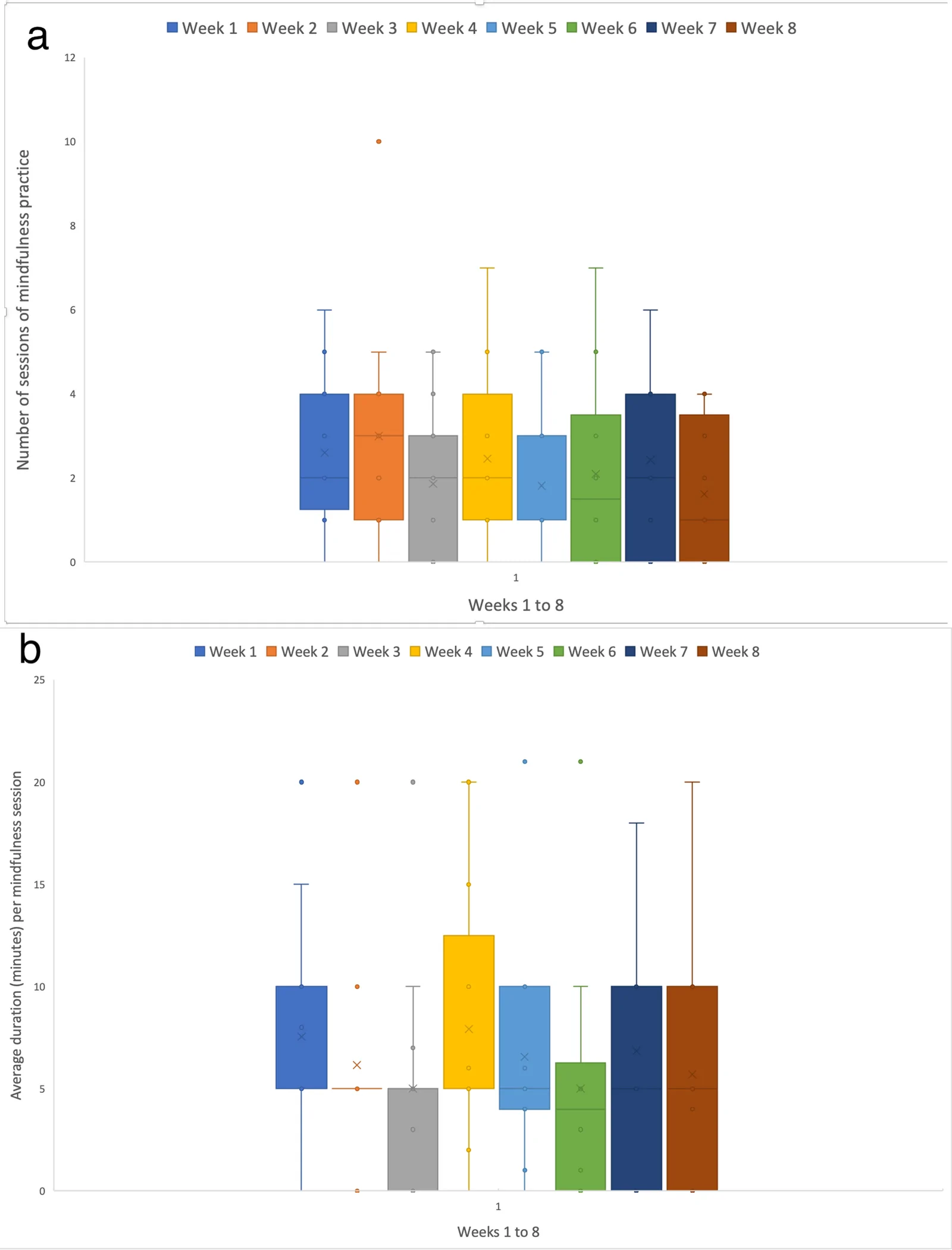
**A** Boxplots of weekly formal mindfulness meditation practice – total number of sessions. **B **Boxplots of weekly formal mindfulness meditation practice – session duration estimated in minutes

Intervention participant adherence to formal mindfulness practice as measured by the MAQ is reported in Fig. 3a and 3b. The boxplots in Fig. 3a show that the median number of sessions practised each week by participating junior doctors across the 8-week program was two to three sessions. Figure 3b shows that the median duration of mindfulness practice remained relatively stable at five to seven minutes per session over the 8-week program. This applies to *n* = 21 participants who returned at least one MAQ survey during the 8-week intervention period.

For each weekly boxplot, the two extreme end points represent the minimum and maximum values of the data series, while the rectangle represents the three quartiles: Q1 (or 25th percentile), Q2 (median), and Q3 (75th percentile). The “x” sign (usually located inside the rectangle) represents the mean value.

For each weekly boxplot, the two extreme end points represent the minimum and maximum values of the data series, while the rectangle represents the three quartiles: Q1 (or 25th percentile), Q2 (median), and Q3 (75th percentile). The “x” sign (usually located inside the rectangle) represents the mean value.

The average rating for formal, informal and overall quality of mindfulness practice was also measured by the MAQ and reported as descriptive statistics in Table 6 together with the average total duration of formal practice. Overall, the median quality of practice was low.

**Table 6:** Summary descriptive statistics for Mindfulness Adherence Questionnaire (MAQ) across the 8-week program for the intervention group (*n* = 28)

Variable	Minimum	Median	Maximum	Mean	Std Dev	Std Error
MAQ_AveQUAN	0	3.9	95.3	8.9	18.0	3.4
MAQ_aveQUAL	0	10.1	43.3	14.7	14.2	2.7
MAQ_aveQUALform	0	4.1	16.5	5.9	5.7	1.1
MAQ_aveQUALinf	0	6.9	26.8	8.9	8.5	1.6

MAQ_AveQUAN: Average Quantity of total formal mindfulness practice per week (in minutes)

MAQ_aveQUAL: Average Quality of formal and informal mindfulness practice combined (Maximum possible total score of 60, from Questions 3–12, MAQ (68)

MAQ_aveQUALform: Average Quality of formal mindfulness practice (Maximum possible total score of 24, from Questions 3–6, MAQ (68)

MAQ_aveQUALinf: Average Quality of informal mindfulness practice (Maximum possible total score of 36, from Questions 7–12, MAQ (68)

**Fig. 4:**
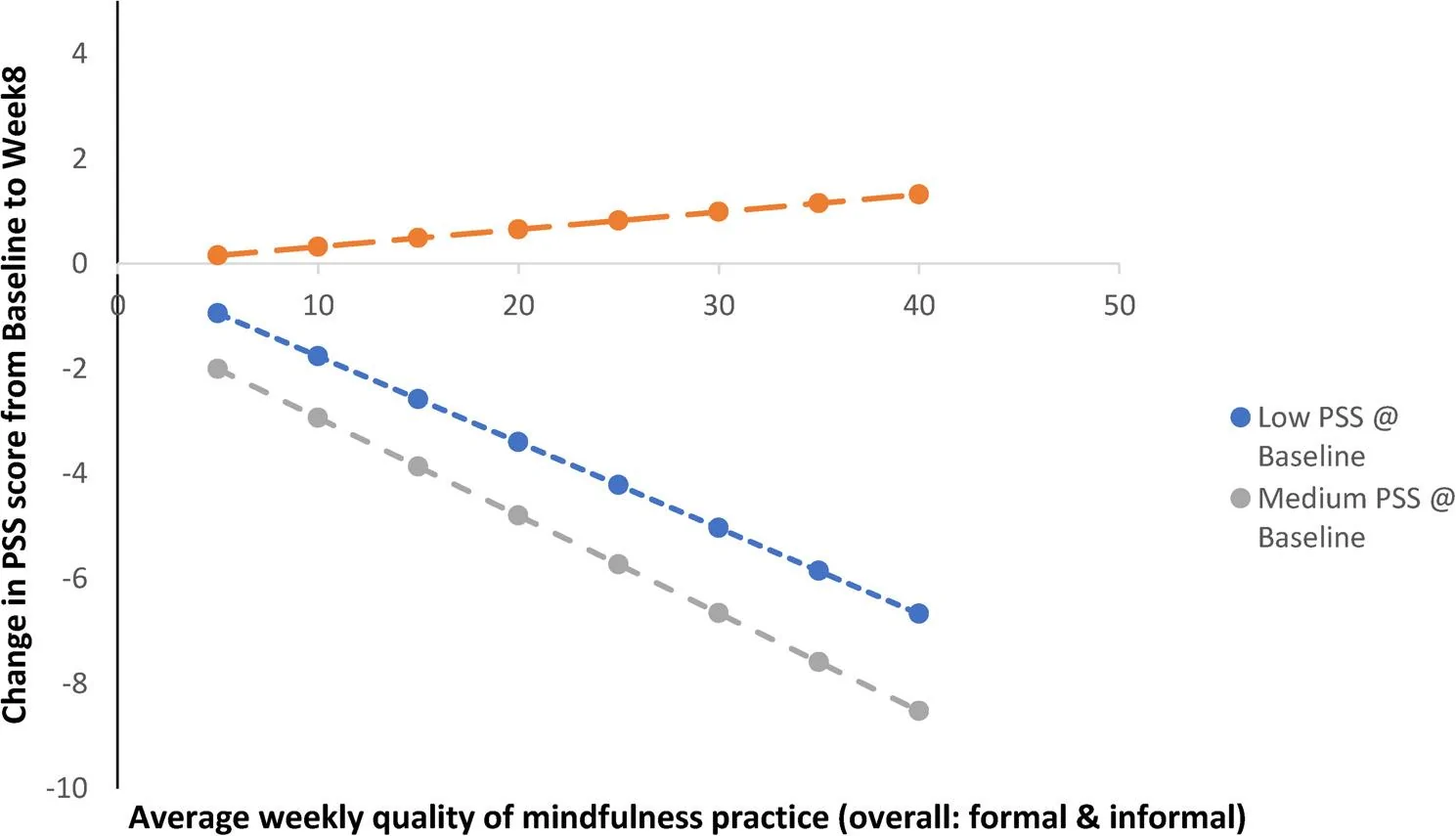
Analysis of Covariance for PSS Score Change from Baseline to Week 8 against weekly quality of mindfulness practice, categorized by PSS levels at baseline. Negative score change indicates increased/ worsened stress level at Week8

None of the four measures within the MAQ were statistically significant in predicting the primary outcome measures. Nevertheless, a pattern was observed for the relationship between the quality of informal practice and perceived stress, measured by the PSS score change, dependent on the participant’s baseline stress level. Specifically, for those with a high level of baseline stress, the quality of informal practice was not associated with changes in the PSS at T2, whereas for those with a low or medium level of baseline stress, the quality of informal practice seemed to be associated with worsened stress levels at T2, although none of these reached statistical significance. Figure 4 provides an illustration of this association.

### Qualitative findings

Individual interviews and focus groups lasted an average of 30 min (range: 9 to 65 min). Participants who enrolled in the program but did not engage fully with the content generally had shorter interviews.

Thirteen intervention group participants, representing half of this group, took part in focus groups and one-on-one interviews.

Table 7 describes the characteristics of the intervention group participants who took part in the focus groups (*n* = 8) and individual interviews (*n* = 5). All junior doctors interviewed were interns.

**Table 7:** Interview and focus group participant demographics

Participant ID	Male/Female	Age (years)	Type of interview
II1 2022	Female	23	Individual
II2 2022	Male	27	Individual
II3 2022	Male	29	Individual
FGI4 2022	Female	26	Focus group
FGI5 2022	Male	24	Focus group
FGI6 2022	Female	25	Focus group
II4 2023	Male	25	Individual
II5 2023	Male	25	Individual
FGI1 2023	Female	25	Focus group
FGI2 2023	Male	23	Focus group
FGI3 2023	Female	26	Focus group
FGI7 2023	Female	25	Focus group
FGI8 2023	Female	36	Focus group

Our qualitative analysis revealed several themes in relation to the barriers to and benefits of participating in an online mindfulness training program randomised controlled trial for junior doctors. The analysis highlighted that although all interviewees gained some benefits from participating in the program, which was not captured in the quantitative findings, they also encountered significant challenges.

1. *Benefits gained from participating in the online mindfulness training program*.

A range of benefits resulting from watching the teaching videos, practicing the mindfulness meditations, and incorporating informal mindfulness into their daily lives were identified. These benefits related to (i) improved self-awareness and emotion regulation (ii) increased self-compassion (iii) productivity at work, (iv) improved professional relationships, and (v) improvements in personal life. Corroborating quotes for each theme have been included in Table 8.

i. Improved self-awareness & emotion regulation.

A number of participants reflected on how their participation in the program resulted in an increased awareness of their emotional state, which improved their capacity to manage stress, overwhelm and anxiety, leading to better emotion regulation. Some participants described how paying closer attention to their emotional and mental state throughout their day provided them with insights into how their emotions affected their performance at work, as well as in their personal life. Several described how they were able to recognise, experience, acknowledge and accept the difficult feelings they experienced while at work, and consciously choose to relax, move to a private space if possible, and focus on the breath while the emotion passed more quickly than previously. One participant described how they would ask themselves “why is my heart beating so quickly? Why is my voice shaking?”, which helped them notice how their physical sensations reflected their emotional state. One participant described how this process provided them with an opportunity to reflect on what had triggered their nervous feelings and what they could do differently if a similar situation arose in the future. Another reflected on how practising mindfulness prevented them from spiralling into anxiety about calling in sick and letting their teammates down, by putting things into perspective and helping them realise that the situation isn’t that bad. Some participants shared how the program served as a reminder to put their mindfulness skills, which they had learnt about at university, into practice during their busy days working as an intern.

ii. Increased self-compassion.

A number of participants reflected that learning about how self-compassion was beneficial to their wellbeing helped them to manage their emotional response to feeling overworked and under-appreciated. One participant described how they came to appreciate that being kind to themselves prevented them from becoming depressed after having received criticism from senior colleagues and another commented that they used to be very self-critical, but after watching the videos on self-compassion they realised that it is not helpful, allowing them to be less hard on themselves and kinder to others.

iii. Increased productivity at work.

Several participants shared how the MTP assisted them with time management, prioritisation of multiple tasks, doing one task at a time, managing uncertainty and overwhelm, and developing critical thinking. One participant explained how practising mindfulness allowed them to notice when they were feeling overwhelmed and take a step back so they could think about what they needed to do now and what can wait, focus on the most important job and do it properly, then move onto the next task. Some participants reflected on how they used mindful breathing and effective task switching to manage their feelings of anxiety, reduce procrastination, stay focussed, and work through their long list of jobs in a strategic manner. One participant described how being mindful led them to question and reflect on the reasoning behind the tasks they were being requested to perform, rather than just doing what they had been asked to do, with the aim of developing their critical thinking skills. Another participant commented on how they felt more relaxed and more productive when taking a mindful approach to their work.

iv. Improved relationships with patients and colleagues.

Some participants commented on the impact that the program had on the quality of their work relationships. Several participants described how practising mindful communication allowed them to be more present with patients and connect with them such that they felt heard and acknowledged, which they believed resulted in better patient care. One participant described in detail how practising mindfulness provided them with better skills for managing ward rounds with multiple doctors and medical students. The participant attributed their MTP participation to developing the capacity to manage complex situations and improve their problem-solving.

v. Improvements in personal life.

A few participants described how their participation in the MTP led to greater emotional awareness of their responses to stressful life experiences and in personal relationships. One participant reflected that practising mindfulness allowed them to leave their work stress behind at the end of the day, which led to less frustration with their partner at home. Another participant provided an example of how practising mindfulness helped them to manage the stress of moving house.

**Table 8:** Benefits gained from participating in the mindfulness intervention

Theme	Quotes
i. Improved self-awareness & emotion regulation	*“I think*,* this is a big one*,* I think probably it’s made me realise how important self care and mindfulness … is to be able to function well … I think it does make you pay more attention to your mental wellbeing and how that’s gonna affect your performance and affect your ability to*,* I guess*,* also segment work from personal life and you know other worries as well. So I think as well it’s probably also helped with anxiety and feelings of stress and feelings of being overwhelmed.”* *Participant FGI3*,* 2023*
	*“I’m quite an anxious person and when I get anxious*,* it manifests as shortness of breath. So there was one video in particular*,* or was it a practice*,* that said to breathe in but then breathe out longer than what you’re breathing in for. I’m sure everyone has things that work for them*,* but that really worked for me and I still practice it today when I get anxious.* *Participant FGI4*,* 2022*
ii. Increased self-compassion	*“I was*,* like*,* more conscious of noticing when I felt really stressed and overworked*,* and it just prompted me to remind myself that we can’t do everything. We’re working in a short staffed workforce and we can all only just do the best that we can. So I guess just being kinder to ourselves and stop telling ourselves that we’re not doing well enough because*,* I mean*,* we’re only interns. I’m only an intern*,* so everything doesn’t rest on us.”* *Participant FGI6*,* 2022*
	*“I guess with regards to my attitudes*,* I think that has changed a little. I think I used to be very hard on myself and used to be very self critical. Listening to the videos of how we should not be too hard of ourselves*,* how we should try not to compare ourselves*,* recognizing that others are also actually in the same state as me*,* that helps the attitude that I have.”* *Participant II2*,* 2022*
iii. Increased productivity at work	*“I guess you could say that*,* specifically*,* like when I do feel like I’m just losing the plot a bit at work and … I just take a minute to focus*,* probably in that regard*,* I’m better at time management ‘cause I can just be like “alright*,* what do I need to do? Just one thing at a time. Let’s just do this now kind of thing.” So yeah*,* that’s probably helped with my time management*,* rather than trying to do like four different things at once and doing them badly.”* *Participant II3*,* 2022*
	*“I think as well when you’re overwhelmed you tend to get very stressed about everything you have to do*,* where I think if you kind of take the moment with the mindfulness to actually think things through properly you kind of have this acceptance of you know that you’re not going to get everything done*,* but you can prioritize what you need to get done first and so you kind of can take a second to really look at what needs to be done*,* you can prioritize. I think that’s also part of competence of being a doctor as well*,* just being able to think things through a bit better.”* *Participant FGI3*,* 2023*
iv. Improved relationships with patients and colleagues	“*So I feel like I have more time with them if I just give them a couple of moments of undivided attention. So I think I’ve been able to work on that doctor patient relationship a bit more than say*,* without the mindfulness program….I mean*,* personally I got into medicine for patients and for people and for the community and doing all that study and all that stress with intern job*,* you can always forget that there is the people aspect to medicine and then of course for the patient*,* they wanna feel heard and listened to and acknowledged and giving them that undivided attention and clear communication only aids that task.”* *Participant FGI4*,* 2022*
	*“The one thing I would probably attribute to the mindfulness program would be communication. I think my communication*,* just being in the moment*,* being with patients*,* being with colleagues. I guess I’m more aware of the situation*,* what I need to get out of it*,* so I’d say my communication has become a bit more clear.”* *Participant FGI4*,* 2022*
	*“So I think being part of this [program] has like given me…an added like extra step in my like processing of my brain between like when I interpret something and react to it and then there’s like a 3 second gap and then my reaction to it. Whereas before it would have been like all quite immediate and probably I wouldn’t have been the most helpful to the medical students. I think I would have still been nice to them*,* but I might have just said*,* hey*,* like*,* can you*,* I don’t know*,* can you please let the juniors in? We just wanna hear what the plan is to write it down. But I think this time I was able to actually explain how the round actually functions and like*,* give them a solution*,* be like*,* hey*,* do you guys wanna split up half of you follow one junior or half of you follow the other one so we can all fit*,* we can all see. Yeah. I just think there was more brain power being used in like it was just all calmer in general…. So yeah*,* I think the mindfulness helped me with that.”* *Participant FGI1*,* 2023*
v. Improvements in personal life	*“I’ve just moved houses and that was quite busy and stressful for me as well*,* but it’s definitely like*,* improved my stress levels overall*,* being more mindful.”* *Participant II1*,* 2022*
	*“I would probably say if it [the mindfulness program] was impacting the way I am with other people*,* it’s being less frustrated with my partner at home and*,* like*,* not taking my work attitude home with me.”* *Participant FGI6*,* 2022*

2. *Challenges from participating in the online mindfulness training program*.

Most of the interviewed junior doctors revealed that they experienced challenges completing the prescribed mindfulness practices and weekly surveys as part of the online mindfulness program study. These related to issues of (i) work stress, (ii) limited time to complete the program, and (iii) lack of satisfaction with some elements of the mindfulness program. Corroborating quotes for each of these themes have been included in Table 9.

i) Work stress.

Practising mindfulness in the context of a stressful work environment was difficult for many participants. Almost all participants reported that their experience of working as a junior doctor at a tertiary hospital was very stressful, describing numerous stressors that impacted on their capacity to engage with the mindfulness program. These included a heavy workload, long shifts, working overtime, lack of supervision by senior doctors, lack of protected time for breaks and having to care for very unwell patients.

ii. Time.

Most participants mentioned that their busy work schedules and limited time for self-care activities, made it difficult to prioritise daily formal mindfulness practice. One participant commented that they didn’t even have time for lunch most days, so to sit down and practice mindfulness was a challenge. Another participant mentioned that being busy at work made it difficult for him to get into a routine with his formal mindfulness practice, which lead to him not practising regularly. Another participant reflected that their own lack of time management skills was a barrier to participating in the program. Some participants explained that in the limited time they had for self-care outside of work, they preferred other activities to practising mindfulness formally, such as going to the gym or watching TV. Additionally, a few participants reported that they found it difficult to make time to complete the weekly research study surveys in addition to baseline and end-of-program surveys due to their heavy workload.

iii) Lack of satisfaction with some elements of the mindfulness program.

A few participants described how they felt bored or irritated doing the same mindfulness practice each day of the week, resulting in them seeking alternative meditation practices or not practising at all. Technical issues related to the online platform limited the engagement with program content for some. Some participants experienced difficulties accessing the Sound Cloud audio files, while others described an aversion to using a WhatsApp group due to its association with work-related tasks. A few participants reported that they felt quite isolated during the program, feeling disappointed that they didn’t have the opportunity for peer sharing. One participant suggested that creating an online community would have enhanced the program experience further.

**Table 9:** Difficulties prioritising mindfulness practice and research surveys

Theme	Quote
i. Work stress	*“With my surgical term*,* we didn’t have an RMO on our team*,* which meant that … you go through the ward round in the morning*,* you’re expected to know everything that’s going on. And then the registrar and the fellows and the consultants*,* well*,* the consultants aren’t even there in the first place. But the registrar and the fellows go away. And you do not see them for the rest of the day.”* *Participant FGI2*,* 2023*
	*“On my surgery rotation*,* often I would be working 10 to 15 h over time*,* but my shifts would be*,* as we discussed previously*,* from 7am til 6pm*,* of which you’d usually work*,* let’s say 6:45 often until 8 or 9 at night*,* and then there would be shift changes from where you would start on an evening shift*,* then go on a run of days that say that would be from 1:00 PM to 10:00 PM. You’d never finish at 10:00 PM and then you start at 6:30 the next day. So often*,* knowingly getting like 3–4 h sleep on those nights. And it was very like I’m still to be honest*,* a little bit burnt out from that experience and also being very stressful and under supported.”* *Participant FGI8*,*2023*
	*“I think in certain teams where there’s not a culture of looking after yourself or there’s not a culture encouraging someone to look after their basic needs*,* you can feel like a robot and you won’t necessarily do those things.”* *Participant FGI8*,* 2023*
ii) Limited Time	*“Being busy being at work*,* I guess*,* yeah*,* I guess I never really got into a routine*,* like when I was gonna do it like. I think when I was starting I was*,* yeah*,* it was easy when I was at home everyday*,* cause I’d just be like “Oh yeah*,* do some mindfulness now*,* I haven’t got anything else to do.” But when I’m starting work*,* I’m up early or I’m getting home and when I’m at home*,* that’s just like I’m just gonna zombie in front of the TV now. Or*,* like*,* I’ve got this on and then you can’t just*,* it [mindfulness] just doesn’t really happen.”* *Participant II3*,* 2022*
	*“I did find the survey data a little bit*,* like the baseline one and the endpoint one which I still need to do. But I’m fine doing those ones because they do take a bit longer and appreciate that. I think I found some of the survey questions when I was going through*,* it was almost like a little bit long for me that I probably put it off because I knew it was going to take a little bit more time until I could be present and actually give my attention to that.”* *Participant FGI8*,* 2023*
	*“And then when I came home*,* I didn’t like putting too many things on my To Do List ‘cause my brain was just like gone already. So then I would pick either going to the gym or doing this [mindfulness]*,* and I always picked going to the gym. I liked it better*,* I think*,* and like because and there was no short-term gratification from doing the mindfulness practice like*,* on the day at the time*,* I would be like*,* it felt like I wasn’t really gonna gain anything from it on the day*,* but obviously I know this is like a long-term thing and it helps you over a long period of time. But then I would pick going to the gym because I’d feel so much better instantly.”* *Participant FGI1*,* 2023*
iii) Dissatisfaction with elements of the mindfulness program	*“But there’s something about*,* and this is something*,* obviously there’s no work around to*,* there’s something about WhatsApp. When I see a WhatsApp notification*,* it just gives me a guttural reaction of “oh God*,* I have to do something.” Because WhatsApp to me is work only. That’s how our bosses communicate with us*,* you know. And that’s how things are being said. And I think when I’m at home*,* I just do not want to see that app at all. Umm*,* so that was … another thing that was sort of a barrier to me as well as all of the stuff which was essentially ours. I felt like I had to choose to either you know*,* foster*,* this*,* this interpersonal relationship*,* or engage in the mindfulness because I sort of know that I need to reserve about an hour and a half each day for just leisure time before I sleep. So that’s just another*,* that WhatsApp spiel was just another thing I wanted to add.”* *Participant FGI2*,* 2023*
	*“And then*,* my personality is funny in that I can get bored quite easily as well. So I often found by the end of the week doing the same mindfulness practice bored me. And I would want a little bit of variety or just something different to keep me going. There was a few times near the end of the week where it got to Thursday or Friday and I just couldn’t bring myself to do the same meditations. I would go onto Smiling Mind [app] instead and pick a different meditation to do*,* just something to keep it interesting or just to suit my mood a bit better.”* *Participant FGI4*,* 2022*
	*“It just would have been really nice to have someone else to talk to about the program as well who was also doing it*,* I think. I think I felt like I was just doing it just alone. I saw a lot of people doing it*,* but I don’t know who these people are. They just look like numbers on a WhatsApp group.”* *Participant II1*,* 2022*

Overall, intervention participants reported a number of benefits but also many challenges to participating in the online MTP as junior doctors. Despite this, most who engaged with the program stated that they would recommend the program to their colleagues. They reflected on the many benefits of junior doctors participating in such a program, including the educational value and the skills they would develop, including that of emotion regulation and stress management.

*“Yeah*,* I think I would definitely recommend it to other people just because*,* like*,* stress management is such an important thing in surviving the first years as a junior doctor. And I think mindfulness is really*,* like*,* one of the like*,* one of the* *best tools* *we can develop to handle that kind of stress.” Participant FGI5*,* 2022*.

## Discussion

In this mixed-method RCT of a tailored online mindfulness training program delivered to junior doctors at two hospitals in Western Australia, we did not observe any quantitative benefits of the intervention in any of our chosen outcome measures including trait mindfulness, perceived stress, self-compassion, occupational stress or work engagement. On the contrary, we observed a statistically significant increase of a modest magnitude in the perceived levels of stress in the intervention group junior doctors who had received the MTP at program completion. We also observed a statistically significant reduction in their work engagement at the final follow up. There were no significant differences between the three time points within the control group for any of the measured outcomes, suggesting these changes were not simply due to potential increases in perceived stress or reduction in work engagement that may occur naturally over time within the junior doctor cohort. Further, there were no significant differences in between-group comparisons for any of the outcomes.

In contrast, qualitative analysis of the trial participants’ reflections revealed reports of several important benefits of the online MTP, including improvements in self-awareness and emotion regulation, self-compassion, work productivity, and professional and personal relationships through participation in the program.

Our quantitative findings cannot be explained by the relatively low number of participants, nor by missing data or loss to follow-up, as our findings did in fact detect a significant, albeit negative, within-group change in perceived stress and work engagement in the intervention group, suggesting that our study was not underpowered to detect change.

Neither does the design of the MTP explain our quantitative findings. In our recently published RCT of the MTP containing similar mindfulness content and the same dose of formal mindfulness, which we conducted with 114 Australian medical students, we reported a significant improvement in measures of trait-mindfulness, self-compassion and study engagement for the intervention group from baseline to program completion, with improvement in trait-mindfulness maintained at 6-month follow-up [56]. In the medical student study, there were no significant within-group changes for any of the outcome measures for the control group, including perceived stress levels and study engagement at program completion or 6-month follow-up.

Given that the baseline perceived stress and work and study engagement scores across our two studies of the MTP were similar for the medical student and junior doctor cohorts, this difference in findings becomes of even greater interest. One explanation for the increase in perceived stress and reduction in work engagement found in the junior doctor study is that although both groups received the same mindfulness intervention, adherence to the program was different between the two groups. Compared to the medical student cohort, the junior doctor cohort spent much less high-quality time engaged with the prescribed formal and informal practice [56].

While qualitative feedback suggests that the online MTP was perceived as well designed for purpose by the junior doctors, there are several explanations as to why the junior doctor group were less likely to engage with the program, including differences between the hospital work environment and the university setting.

Most junior doctor participants in our study reported high-stress, time-poor work settings, and reported challenges with finding time to practice their mindfulness. In addition, it is conceivable that taking part in the study itself as an intervention participant, including the requirement to practice mindfulness daily and to complete the required surveys, may have itself compounded existing stress levels, especially in intern participants who were just commencing their hospital work.

Further, the reduction in program adherence during the second half of the intervention, which included training in attitudinal skills, may have resulted in participants not developing some facets of mindfulness as much as others. For example, junior doctors’ self-awareness of their mental state may have increased in response to attention regulation training during the first half of the program without sufficiently integrated attitudinal training, such as non-reactivity, non-judging and acceptance. Development of attitudinal skills with mindfulness practice typically requires more time and repetition than development of attention regulation skills in order to achieve comparable results [73]. The dose of attitudinal skills training received by the participants may have been insufficient to positively affect outcome measures such as perceived stress and work engagement, considering the degree of work stress levels to which the junior doctors were exposed. A longer follow-up period of greater than one-month may also have allowed more time for these attributes to develop, consistent with da Silva’s suggestions that the effects of mindfulness practice are cumulative and may take time to manifest as measurable outcomes [27].

Our findings reflect the effects of the MTP without influence from facilitator intervention or formalised peer sharing. However, it is also plausible that the lack of these elements as part of the MTP resulted in increased self-awareness by junior doctors of their mental state thus increasing their perceptions of stress, without access to in-program support. Further, junior doctors reported in our interviews that they would have appreciated the opportunity to speak to other participants about the program. One of the barriers to practising mindfulness reported by study participants included boredom with the repeated guided meditations, which may have been mitigated by a facilitator putting such experiences into perspective or by receiving support from peers. Other mixed-methods studies of online MBIs that have included opportunities for junior doctor participants to interact with course facilitators and their peers have reported significant changes in outcomes, including trait-mindfulness, mental wellbeing and perceived stress [53–55]. Qualitative findings from two of these studies have reported specifically on the value of sharing clinical experiences with colleagues, which may have contributed to the effectiveness of these interventions [53, 55]. The importance of person-to-person connection in mindfulness interventions designed for junior doctors is also strengthened by the findings of in-person MBI studies that have reported positive changes in trait-mindfulness post-intervention [33, 37, 43, 46, 47].

Further, given the relative recency of research into online MBIs, there remains controversy as to the ideal outcome measures and duration of follow-up to utilise in such work. It is important to acknowledge that assessing perceived changes in psychological distress and wellbeing symptoms are in general complex and subjective [27]. In addition, Kaisti [65] suggests that the effects of mindfulness interventions may be difficult to capture with self-report outcome scales at all, since they may not assess qualitative and evolving phenomena that occur during or after the mindfulness intervention is complete. This would be particularly relevant to this group of very busy junior doctors whose levels of stress would be constantly changing in response to their specific work situation. Further, the effects of mindfulness practice are cumulative, taking time to manifest as measurable outcomes [27]. Taken together, these may provide another explanation for the lack of positive findings in our study.

Another potential explanation for our unexpected null and negative findings lie in the phenomenon of a ‘response-shift’ rather than inappropriate outcome measures and follow-up. A ‘response-shift’ occurs when changes in internal standards, values and conceptualisation decouple true change in health status from measured scores on a fixed questionnaire [74]. Mindfulness interventions have been reported to be associated with the response-shift phenomenon [75] and have been detected using a ‘then-test’ as part of mindfulness program RCT evaluations. ‘Then-test’ questionnaires are generally administered together with post-intervention surveys requiring participants to reassess their baseline survey retrospectively, with the same frame of reference, internal standards and values as they have at the time of the post-survey. Comparison of the baseline, then-test and post-test survey scores allows for quantification of potential confounding response-shift bias [76]. After adjusting for the response-shift, the effect size of mindfulness-interventions appears to be larger, implying that pre-post survey scores underestimate the actual changes in health status [76]. Thus, it may be possible that our study underestimated the true effect sizes of change in outcomes with the intervention. Were these to have been adjusted with a then-test, we may have detected positive changes or lack of negative changes in survey scores.

The findings of this study, embedded within the participants’ description of their work environment, highlights the important risk of harm when individually targeted wellbeing initiatives are introduced in a hospital system that does not provide protected opportunities to participate, resulting in participants feeling overwhelmed rather than experiencing the expected benefits. Further, it has the potential to be harmful to already stressed junior doctors who already have high expectations of themselves. As discussed in a systematic review of workplace interventions conducted to improve wellbeing in healthcare professionals, organisational interventions that target work conditions and prevent occupational stress, although usually more difficult to implement, are better at promoting and maintaining wellbeing amongst healthcare professionals than interventions designed for individual doctors [77]. Given the findings of our research and systematic reviews, the obligation of healthcare institutions is to implement wellbeing interventions at an organisational level that facilitate safe engagement with programs such as the MTP.

Despite this, our findings reveal that some junior doctors in the intervention group did experience a range of benefits from participating in the MTP and would recommend the intervention to their colleagues. Thus, we propose that such interventions continue to be trialled and organisations be encouraged to find ways in which such wellbeing interventions can be more accessible to doctors within their workplace to allow all participants to achieve the benefits.

Our study has a number of strengths. We utilised a rigorous RCT design and recruited an appropriate sample of junior doctors to evaluate the impact of previously trialed MTP using a mixed-methods approach. We included a range of wellbeing outcome measures relevant to junior doctors. Further, follow up data was obtained from over 60% of participants. There were, however, also several limitations. We did not include another intervention comparator group and the ‘usual curriculum’ comparator does not control for attention, expectation or time spent in structured self-care activities, which may have affected the outcome measures. Follow-up only extended to 3-months post randomisation. Further, our study was conducted in two hospitals within a tertiary health service, limiting the generalisability of our findings. Additionally, although overall trait-mindfulness did not change, individual mindfulness facets were not measured, which made it difficult to ascertain if some facets of mindfulness improved while others did not.

### Implications for future research

We have a number of suggestions for future studies in the area of mindfulness interventions based on our trial. Participant engagement is critical to the success of any mindfulness intervention. We suggest that before any mindfulness intervention is trialled, the likelihood of participant engagement is assessed and optimised. Future studies could utilise clinical trial development frameworks, such as the ORBIT Model for Developing Behavioural Treatments for Chronic Diseases, to guide the refinement of novel online MBIs as well as the trial protocol to ensure maximum participant engagement and therefore mindfulness practice [78]. The ORBIT Model suggests four phases of behavioural treatment development, including a design phase, preliminary testing, efficacy trial and effectiveness testing, that allow for flexible, careful and progressive research development [78]. Our current interventional RCT would have benefitted from a preceding feasibility or efficacy pilot trial in the junior doctor group, similar to that carried out in the medical student participant cohort [57] although we had conducted a less formal feasibility consultation with the relevant hospital medical education unit prior to proceeding with the trial. Our study was also limited by not having an active control comparator, which was a pragmatic decision, driven by budget and time constraints. However, we recommend that future studies of novel online MBIs could utilise a three-arm RCT comparing the intervention to an active control group (such as an in-person MBI) and a waitlist control group. With regards to outcome measures, including facet-level measures of mindfulness would be helpful for better understanding which components of the intervention are driving potential change. Different wellbeing outcome measures, such as quality of life, sleep quality and burnout could be utilised to assess the impact of the MTP. Adding a ‘then-test’ to post-intervention surveys could also be considered to allow for a potential ‘response-shift’. Longer term follow-up six to 12 months post intervention would be valuable, although this remains a challenge in this busy and mobile group of doctors. Future studies of this intervention could recruit participants from different health services and different countries. Finally, it is important to note the importance of conducting formal safety monitoring in studies of online MTPs to ensure that any harms experienced by participants during the course of the study are clearly identified, recorded, and addressed.

## Conclusion

This mixed-methods RCT found that a novel, tailored online MTP was not associated with improvements in outcome measures of trait-mindfulness, self-compassion or occupational stress, however was associated with an increase in perceived stress at program completion and reduction in work engagement at the final follow up for participating junior doctors working in a busy tertiary hospital. This contrasted with our previous study of a similar intervention delivered to medical students, which found modest improvements in trait mindfulness, self-compassion and study engagement for those participants completing the intervention. Our qualitative findings suggest that junior doctors do experience benefits from participating in the MTP and highlight that organisational factors and insufficient in-program support impact on program adherence, which likely affects the efficacy of mindfulness interventions in this population. Optimising work environments and implementing online mindfulness interventions at an organisational level will reduce the expectations placed on individual doctors to maintain their wellbeing in an over-stressed system, allowing them the opportunity to flourish.

## Figures and Tables

**Figure:**
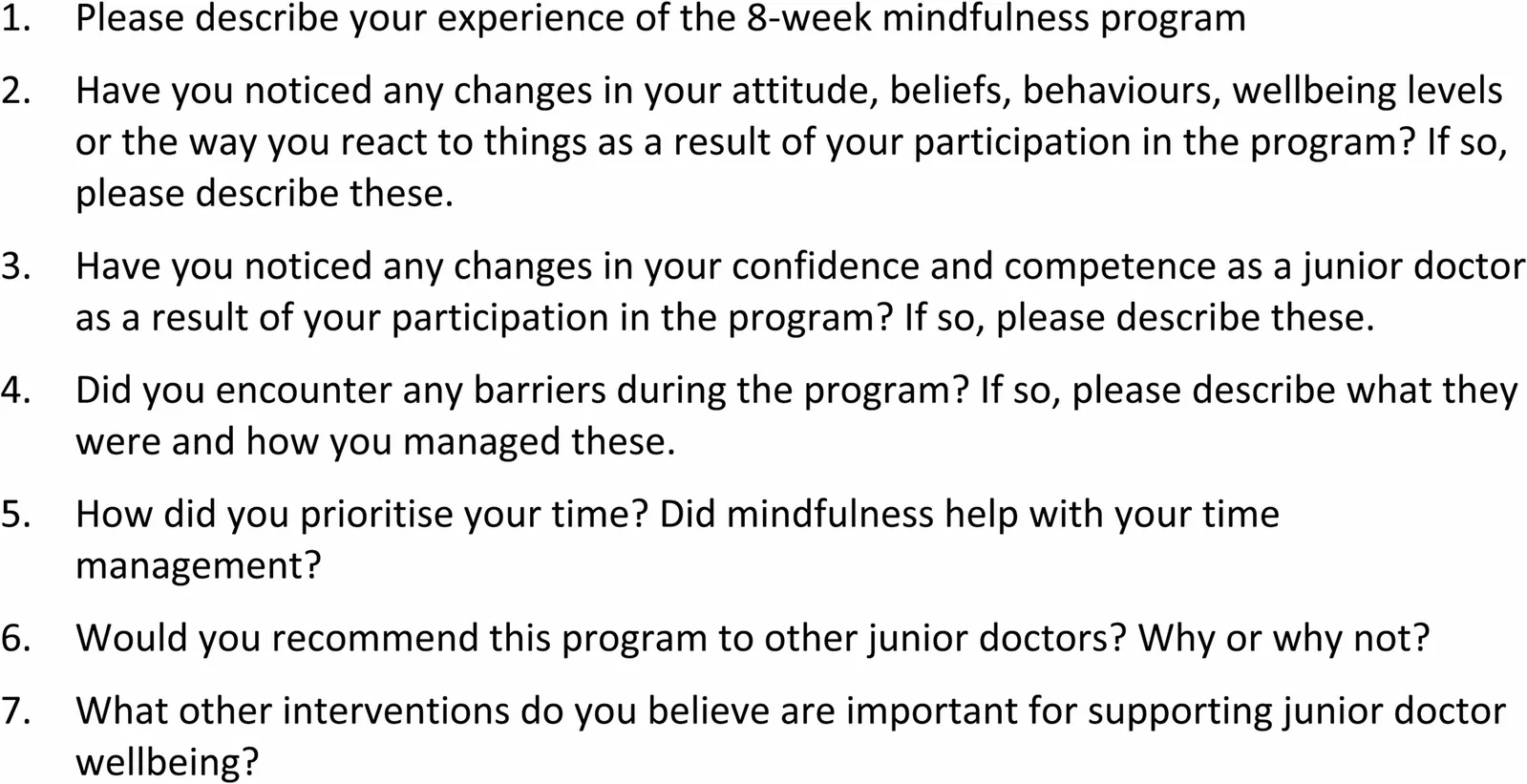
Semi-structured interview questions for Intervention arm focus groups & individuals

**Figure:**
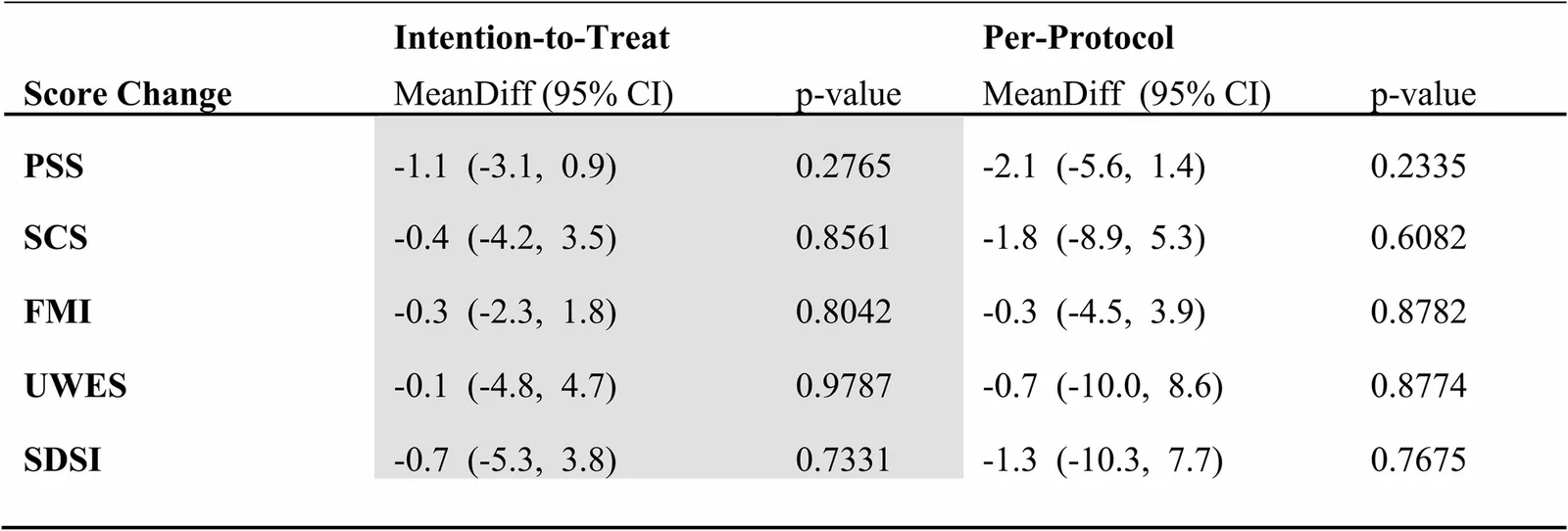
Sensitivity analysis comparing Intention to Treat versus Per-Protocol approaches to analysis of primary outcomes

## Data Availability

The data that support the findings of this study are not openly available due to reasons of sensitivity and are available from the corresponding author upon reasonable request. Data are located in controlled access data storage at the University of Western Australia.
